# Age-related Deficits in Recognition Memory are Protocol-Dependent

**DOI:** 10.14336/AD.2017.1223

**Published:** 2018-10-01

**Authors:** Diano F. Marrone, Elham Satvat, Anuj Patel

**Affiliations:** ^1^Dept. of Psychology, Wilfrid Laurier University, Waterloo, ON N2L 3C5, Canada; ^2^McKnight Brain Institute, University of Arizona, Tucson, AZ 85724, USA,; ^3^School of Public Health & Health Systems, University of Waterloo, Waterloo, ON N2L 3G1, Canada

**Keywords:** recognition memory, aging, hippocampus, perirhinal cortex

## Abstract

The perirhinal cortex (PRh) is a critical mediator of recognition memory, and a wealth of evidence points to impairment in PRh function with age. Despite this evidence, age-related deficits in recognition memory are not consistently observed. This may be partially due to the fact that older animals also have well-established deficits in hippocampal function, and many protocols that assess perirhinal function are also sensitive to hippocampal damage. When using one of these protocols, spontaneous object recognition in an open field, we are able to replicate published age-related deficits using pairs of complex objects. However, when using zero-delay object recognition, a task that is more resistant to the influence of changes in hippocampal function, we find no significant age-related differences in recognition memory in the same animals. These data highlight the importance of the protocol used for testing recognition memory, and may place constraints on the role of the PRh in age-related recognition memory impairment as it is typically tested in much of the literature.

Normal aging is associated with changes in many cognitive functions, including memory. While different types of memory processes involve the participation of a distributed network of brain structures, certain regions can play a more predominant role in one form of memory over another. Identification of these regions helps researchers relate changes in behaviour to neuronal mechanisms. In turn, this understanding is critical to developing therapeutic targets for the promotion of successful cognitive ageing. Within this context, object recognition memory and how it changes over the lifespan has been the subject of intense study.

Work with animal models has focused predominantly on variations of the spontaneous object recognition (SOR) task [1] that capitalizes on rodents’ innate tendency to explore novel objects more than familiar ones. Although many studies report age-related deficits in this task (e.g., [2-14]), others do not (e.g., [12-17]). Thus, our understanding of recognition memory and how it changes with age can be furthered by attempting to resolve this discrepancy in the evidence.

One factor to consider is the contribution of different brain regions to performance under different testing protocols. Although specific brain regions certainly play a predominant role in specific mnemonic functions, memory undoubtedly involves the participation of multiple highly overlapping sets of brain structures. The perirhinal cortex (PRh) has long been considered the primary mediator of object recognition memory due to its role in representing high-order sensory information [18]. There is evidence, however, that the hippocampus is also capable of mediating perceptual learning [19] and may play a role in these tasks, at least under some circumstances (e.g., [20-23]). In fact, the majority of previous work showing age-related deficits in SOR has been done under circumstances in which the hippocampus may contribute - with testing conducted in open fields and with delay intervals during which animals are removed from the environment. This is problematic considering that ageing is associated with deficits in a broad range of hippocampus-dependent tasks [24]. Varying the testing protocol, however, may provide measurements of recognition memory that are more resilient to changes in hippocampal function. For instance, tasks in which animals are removed from the testing apparatus between exposure an object and recognition testing are far more susceptible to impaired hippocampal function [25] relative to tasks in which animals remained in the test apparatus during the retention interval [26]. Moreover, testing in a more confined space with high walls can reduce contextual cues and thus minimize the hippocampal contribution to performance. Testing under these circumstances results in tasks, such as zero-delay object recognition (ZOR), that are sensitive to damage to the perirhinal cortex and not the hippocampus (e.g., [27]). Although these variations have the potential to provide a more accurate assessment of perirhinal function across the lifespan, they have yet to be implemented in testing older animals.

While it has long been known that ageing spares performance in some types of memory tasks and not others, the ZOR and SOR have been presented as equivalent tasks in the sense that they are thought to tax the same memory abilities and object recognition network. More specifically, tasks with at least some of the features of ZOR (particularly having the task occur in a Y-maze and with little or no delay) are commonplace in the “basic” literature on recognition memory, while these types of tests are relatively rare in the context of ageing research. This is only problematic, however, if these variations in testing protocol produce meaningful differences in results. To test the hypothesis that testing protocols affects recognition memory performance in older animals, the current study assessed young (6 months), middle-aged (12 months) and aged (24 months) F344 rats both in SOR and ZOR. Spatial learning was also assessed using the Morris water maze [28].

## MATERIALS AND METHODS

### Subjects

Twenty-nine male Fischer 344 rats (bred from stock originally obtained from Envigo, Indianapolis, IN) were used for these studies, at three age ranges: young (4-6 months, n = 9), middle-aged (11-13 months, n = 9) and aged (23-25 months, n = 11). All animals were housed in standard housing (home cages) under a 12:12 light/dark cycle and were given food and water *ad libitum*. All animals were experimentally naive at the beginning of the testing described here. All procedures were approved by the Wilfrid Laurier University Animal Care Comittee, in accordance with the Canadian Council on Animal Care procedures and guidelines.

All animals were first tested in the Morris Water Maze [28], followed by the zero-delay object recognition (ZOR) task [27], and then in a spontaneous object recognition (SOR) task [1]. Each of these tasks is described below. All animals were handled for 7 days prior to training, and at least 5 days passed between tests. In all tasks, animals were tracked using an overhead video camera and Any-maze software (Stoelting, Kiel, WI, USA).

**Figure 1.**
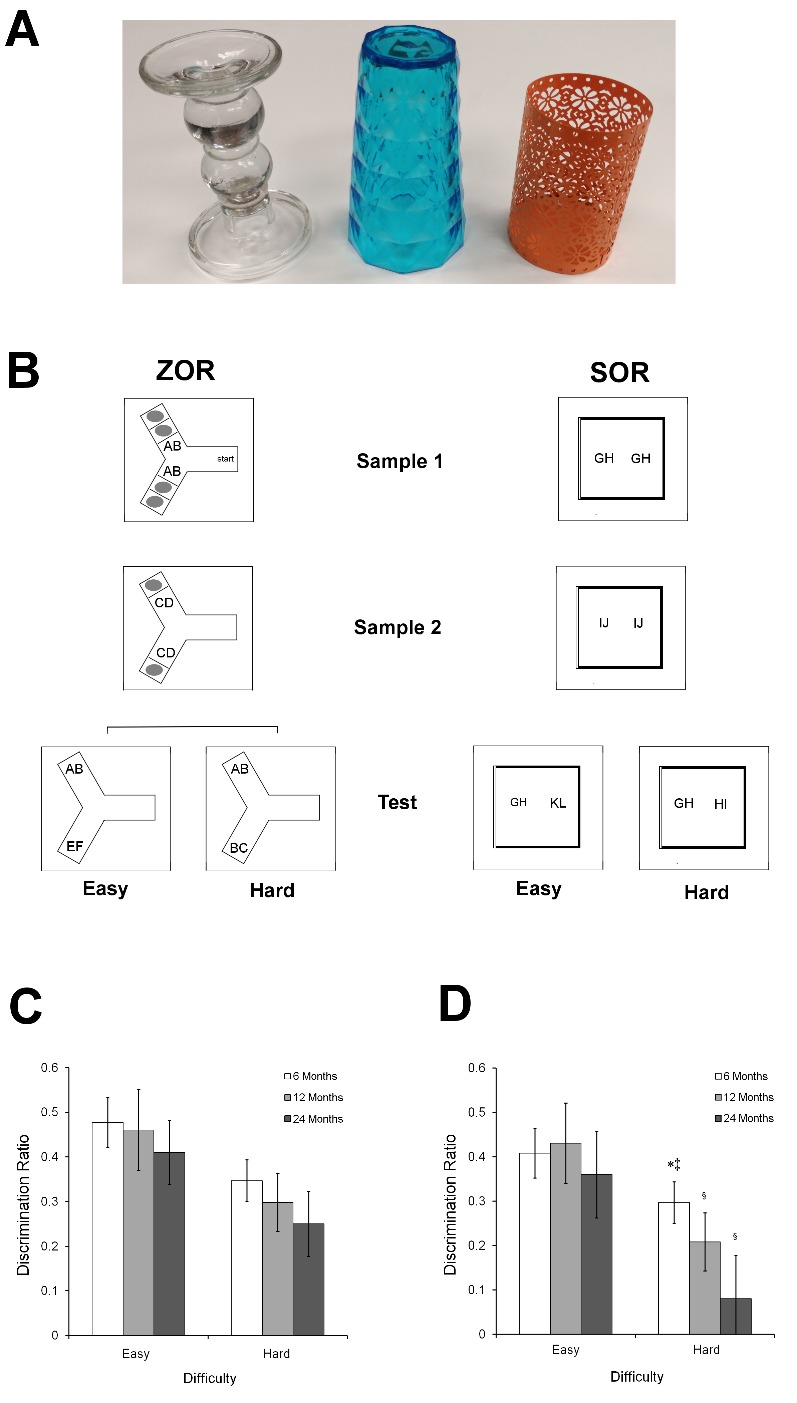
Changes in testing protocol determine the presence of age-related recognition memory deficits Examples of object pairs used are shown **(A)**. All objects were junk items purchased from local stores and included dog toys, children’s toys, and small household decorative items such as candlesticks. The procedures **(B)** for zero-delay object recognition (ZOR, left) and spontaneous object recognition (SOR, right) are depicted. In ZOR, sample trial 1 consists of 2 identical pairs of objects pairs (AB) presented in each arm of a y-maze that remained until the animal had explored one of the objects for at least 25 sec. After this criterion is met, barriers are removed to reveal a new identical pair of objects (CD). Once the rat explored one of these objects for at least 25 sec, the second barrier was removed to reveal a familiar (AB) object and a novel pair of two unique objects (EF, easy condition), or a novel combination of previously seen objects (AC, hard). The SOR task uses similar methodology in an open field. In SOR, sample trial 1 consists of 2 identical pairs of objects (GH) presented within an open field until the animal had explored one of the objects for at least 25 sec. The rat was then removed from the open field for 120 sec, while a new identical pair of object was placed in the field (IJ). The rat returned to the field for sample trial 2 until it explored one of the objects for at least 25 sec. After a 120 sec delay, rats were then tested with a familiar (GH) object and a novel pair of two unique objects (KL, easy condition), or a novel combination of previously seen objects (HI, hard). Quantitative analyses of the ZOR **(C)** and SOR **(D)** reveal different effects obtained from these protocols. While 6-month-old (white bars) and 12-month-old (light grey) animals generally perform well under all conditions, 24-month old animals (dark grey) show recognition memory deficits only in SOR, while their performance in ZOR is relatively intact (data are mean ± SEM, * = p < 0.05 vs 12 months, ‡ = p < 0.05 vs. 24 months, § = p <0.05 vs. easy trials in the same age group).

In the ZOR and SOR, object pairs were counter-balanced between and within animals across both tasks. All objects were junk items purchased from local stores and included dog toys, children’s toys, and small household decorative items such as candlesticks (see Fig. 1A). Investigation of an object was defined by the rat physically interacting with the item (i.e., rearing, biting) or sniffing the object (i.e., nose twitching while oriented toward the objects and within 2 cm).

### Morris Water Maze

In order to assess hippocampal function and to be able to compare this performance with recognition memory, all animals were tested on their spatial memory performance in the Morris water maze (MWM) as previously described [29,30]. Briefly, a 1.83 m diameter circular maze was located in a well-lit room containing prominent visual cues and filled with water at 25 ± 1°C made opaque with tempura paint (Scholar’s Choice, Kitchener, ON). The rats received 6 spatial learning trials per day for 4 days. Rats were placed into the water facing the maze wall at one of four start positions (i.e., North, West, South, and East) at the beginning of each trial. Rats could escape on a 12-cm diameter platform located ~1cm below the water surface. If an animal failed to reach the platform within 120 s, it was guided there by the experimenter. Every trial ended with the rat on the platform for 30 s. Following spatial training, a 60-s probe trial tested retention of the platform location when the platform was removed. This was followed by 6 visible platform trials immediately following the probe trial (i.e., on training day 4), and another 6 visible platform trials the following day (i.e., training day 5). On these trials, the platform was marked and protruded above the waterline. Performance on these trials is intended to assess whether age-related differences in spatial trials can be attributed to deficits visual acuity or motor ability.

### Zero-Delay Object Recognition Task

The ZOR task was conducted as previously described [27] in order to provide a test of object recognition that has been demonstrated as being resistant to changes in hippocampal function. Briefly, animals were habituated to the Y-shaped maze (40 cm long arms, 45 cm high walls) by exposing them to the apparatus for 10 min per day for two consecutive days. Following this, animals were exposed to object pairs under two conditions: an easy condition and a hard condition. Each test consisted of three phases: sample phase1, sample phase 2, and a choice phase (Fig. 1B). All object sets used in a given trial were placed in the apparatus before the rat was placed in the start box. The rat was then placed in the start box with the guillotine door lowered. The guillotine door was then raised to allow the rat into the exploration area of the apparatus. When the rat exited the start box, the guillotine door was lowered to prevent re-entry, and the sample phases began. In the hard condition, two duplicate sets of sample 1 object pairs (AB) were revealed to the rat upon exiting the start box. Although the two pairs were identical, each pairing consisted of two different objects (i.e., a pair made up of a ball and a candlestick was presented alongside another pair of objects made up of a ball and a candlestick). Sample phase 1 ended when the rat had explored any combination of the identical objects for 25 sec. Upon completion of exploration of sample 1 stimuli, the sample 1 stimuli were removed, the door was opened between sample 1 and sample 2, and the rat was immediately shown the sample 2 (CD) stimuli. After exploring sample 2 objects for 25 sec, the stimuli were removed, the door between sample 2 and choice was opened, and the novel and familiar (AB) stimulus pair were immediately presented to the rat in the choice phase for 5 min. The easy condition was identical to the hard condition except that in the easy condition, as a pair of new objects (EF) the rat had not encountered before, while in hard condition, the object was a novel configuration of familiar objects (AC). All rats were tested in both the hard and easy conditions, and the order of testing as well as the objects used were counterbalanced between animals. Each age group’s mean time spent exploring each object pair under each condition is shown in Table 1.

**Table 1:** Mean object exploration time across all conditions

Age (months)	Task	Condition	Novel object exploration time (sec)^1^	Familiar object exploration time (sec)^1^
4-6	SOR	Easy	25.29 ± 4.86	19.29 ± 2.15
		Hard	19.57 ± 2.94	21.19 ± 3.66
	ZOR	Easy	18.75 ± 4.17	19.12 ± 6.81
		Hard	18.01 ± 1.89	17.11 ± 2.42
11-13	SOR	Easy	29.4 ± 5.58	23.2 ± 2.99
		Hard	19.0 ± 2.09	18.4 ± 3.01
	ZOR	Easy	22.29 ± 3.76	18.29 ± 5.05
		Hard	29.1 ± 7.75	24.88 ± 11.40
23-25	SOR	Easy	22.56 ± 3.50	24.11 ± 6.87
		Hard	25.78 ± 7.82	23.1 ± 10.89
	ZOR	Easy	19.45 ± 4.17	18.72 ± 6.81
		Hard	21.09 ± 7.67	21.00 ± 10.54

1 The amount of time spent exploring each object (as defined in the Methods section) within the first 2 minutes of each test trial

### Spontaneous Object Recognition

In order to analyze the effect of the training protocol on recognition memory performance, animals were tested in SOR (Fig. 1A), the most common protocol for testing recognition memory, with several variations in order to make it as consistent with the ZOR protocol as possible. Testing was conducted in an open field (50 x 76 cm) with three black walls and one white wall, each 20-cm high. Testing was preceded by 10 minutes of habituation exposure to the empty apparatus for two consecutive days. Comparable to the procedure for ZOR, rats participated in two sample phases and then two test phases with 2 different levels of perceptual difficulty: easy and hard. Sample 1 object pairs (GH) were placed in the open field prior to placing the rat inside. Sample phase 1 ended when the rat had explored the identical objects for 25 sec. Upon completion of exploration of sample 1 stimuli, the rat was removed and placed in a cage located in the testing room for 120 sec. During this time, the stimuli that were in the open field were changed. The rat was returned to the open field, which now contained the sample 2 (IJ) stimuli. After exploring sample 2 objects for 25 sec, the rat was removed and placed in the adjacent cage for 120 sec while the objects were changed. The rat was then returned to the open field, which now contained a novel and familiar (GH) stimulus pair for 5 min. The easy condition was identical to the hard condition except that in the easy condition, the pair of new objects (KL) were ones that the rat had not encountered before, while in the hard condition, the objects were a novel configuration of familiar objects (GI). All rats were tested in both the hard and easy conditions, with the order of testing and the objects used was counterbalanced between animals.

### Statistical analysis

Performance in the MWM was analyzed using repeated measures analysis of variance (ANOVA), using training day as the repeated factor and age as the between-subject factor. Performance in probe trials was assessed using a one-way ANOVA as well as a paired t-test for each age group comparing time spent in the quadrant of the platform paired with the opposite quadrant. For both object recognition tasks, a discrimination index was calculated for the final stimulus set for each rat as a ratio of the time spent exploring the novel pair (T_N_) relative to the time spent in the familiar pair (T_F_) within the first 2 min of the trial as follows: (T_N_ - T_F_)/(T_N_ + T_F_). Performance in both object recognition tasks was analyzed using repeated measures ANOVA, using condition (i.e., easy or hard) as the repeated factor and age as the between-subject factor. Discrimination index scores from the choice phases were further compared for each age group individually using a one-sample t-test relative to a 0 value (indicating no recognition). A direct comparison between the tasks was also conducted using an ANOVA evaluating age by task (i.e., SOR vs. ZOR) for the hard condition only. All post-hoc tests were conducted with Tukey’s HSD.

In addition, performance in both recognition memory tests was compared to spatial memory performance in the MWM as previously described [31]. Briefly, data from all age groups were combined into a single analysis by converting the discrimination index from the hard condition in both ZOR and SOR and path lengths on the final day of MWM testing into *z*-scores calculated independently for each age group. This normalization effectively permits comparing performance across age groups on all 3 tasks with the number of animals tested [31] and prevents detecting artificial correlations due solely to the differences in the means across age groups [32].

## RESULTS

### Spatial learning in the Morris water maze

**Figure 2.**
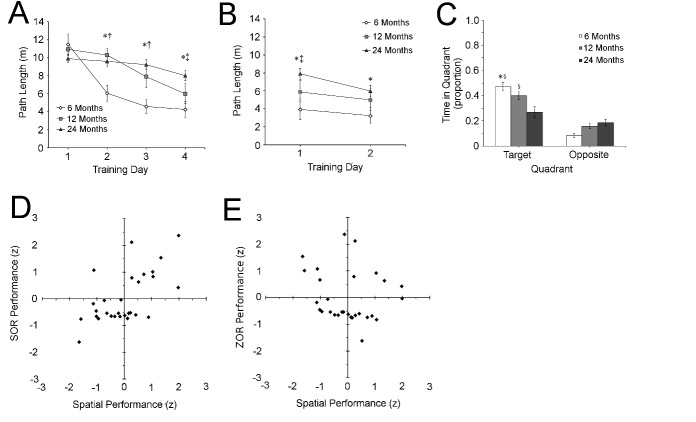
Age-related spatial memory deficits correlate with spontaneous object recognition performance Analysis of path lengths **(A)** in the Morris water maze (MWM) shows that when the platform was hidden, 6-month-old rats (white diamonds) swam shorter paths to reach the hidden platform than either 12-month-old (light gray square) 24-month-old (dark gray triangle) rats by day 2 of training. By day 4, 12-month-old rats also outperformed 24-month old ones. During trials in which the platform was visible **(B)**, all 3 age groups had significantly different path lengths on day one, and this difference became much smaller by day 2 such that only 6-month-old and 24-month-old rats shows a significant difference. During the probe trial **(C)**, both 6-month-old (white) and 12-month-old (light grey) rats spent significantly more time than aged rats (dark grey) in the quadrant that previously held the platform (target) than the opposite quadrant. Regression shows that spatial memory performance does not predict the performance of individual animals in zero-delay object recognition (ZOR, **D**). However, spatial memory significantly predicts SOR performance (**E**) in individual animals (all data are mean ± SEM; * = p < 0.05, 12 vs 24-moth-old; † = p < 0.05, 6-month-old vs 24-month-old; ‡ = p < 0.05, 6-month-old and 24-month-old; § p < 0.05, vs opposite quadrant in the same age group).

Consistent with previous reports (e.g., [13, 29-31]), aged animals showed a deficit in locating a hidden platform in the MWM (Fig. 2).

#### Spatial learning trials

All rats showed performance improvements (i.e., shorter path lengths) over trials (main effect of training day F_1,26_ = 30.60; p < 0.001). A significant main effect of age on path length (F_2,26_ = 3.96; p = 0.04) was also observed, indicating that aged rats took longer paths to the platform. A significant age by training day interaction was also present (F_2,26_ = 13.19; p < 0.001), and *post hoc* analyses confirmed that age-related differences grew larger as training progressed. Six-month-old rats performed significantly better than their older counterparts beginning on day 2 of training, while 12-month-old rats and 24-month-old rats did not significantly differ in their performance until training day 4 (p < 0.05). These data confirm that the aged rats in the present study had impaired spatial memory.

#### Probe trial

Analysis of performance during the probe trial (Fig. 2C) confirmed the age-related deficit in spatial memory observed in the presence of the platform (main effect of age: F_2,26_ = 3.58; p = 0.04). Post-hoc testing showed that 24-month-old rats spent significantly less time in the target platform quadrant than either 6-month-old (p < 0.01) and 12-month-old (p = 0.02) rats. Consistent with this observation, both 6-month-old (t_8_= 14.02; p < 0.001), and 12-month-old (t_8_ = 3.39; p = 0.009), rats spent significantly more time in the quadrant that previously held the escape platform than the opposite quadrant, while aged rats did not (t_10_ = 1.12; p = 0.29).

#### Visible platform trials

Consistent with performance during spatial trials, both young and aged rats showed significant learning in the visible platform trials, assessed by a significant effect of training day (F_1,26_ = 12.17; p = 0.002). A main effect of age was also observed (F_2,26_ = 7.08; p = 0.004), as well as a significant interaction between age and training day (F_2,26_ = 3.54; p = 0.04). Although post-hoc analyses indicate that aged animals took significantly longer than both middle-aged and young animals to reach the visible platform on day 1 (p = 0.01), much smaller differences in the average distance swam to reach the platform were observed on day 2. Consistent with this observation, post-hoc tests showed no significant difference was observed in the distance travelled between aged and middle-aged rats, consistent with previous reports [7, 29, 30, 33], nor between middle-aged and young rats. A significant difference remained between young and aged rats (p = 0.04). Despite this difference, the reduction in path length among old animals and the comparable performance relative to middle-aged rats indicating that aged animals were able to consistently find the visible platform with sufficient training. These data make it unlikely that the age-related differences in spatial trials or subsequent recognition testing are due to deficits in visual acuity, although deficits in motor abilities may be possible, particularly when comparing 6- and 24-month old animals.

### Zero-Delay Object Recognition Task

Despite showing profound impairment in spatial learning, ZOR performance indicated that the recognition memory of aged animals was relatively preserved.

#### Sample Phase

All animals explored during the sample phases for 25 sec in under 5 min on all trials. Analysis of the total time required to complete 25 sec of exploration in the sample phase was analyzed. This analysis revealed no significant difference between age groups, no significant effect of condition (i.e., easy vs. hard), and no significant interaction (F < 1.49; p > 0.22 in all cases).

#### Choice Phase

During the first 2 minutes of the retrieval trial (Fig. 1C), a significant main effect of condition (F_2,26_ = 4.34; p = 0.03) demonstrated that discrimination index scores were significantly lower for hard trials than for easy ones for all 3 age groups (p < 0.05 in all 3 cases). No significant effect of age (F_2,26_ = 2.01; p = 0.15) or age by difficulty interaction (F_2,26_ = 0.23; p = 0.79) were observed. In all cases, animals from all age groups showed discrimination scores significantly different from 0 (chance) during both the easy (young: t_8_ = 3.54, p < 0.01; middle-aged: t_8_ = 3.31, p < 0.01; aged: t_10_ = 3.17, p = 0.01) and hard (young: t_8_ = 2.86, p = 0.02; middle-aged: t_8_ = 2.51, p = 0.03; aged: t_10_ = 2.29, p = 0.04) recognition tests.

### Spontaneous Object Recognition Task

Consistent with previous reports (e.g., [7,12,14]), age-related differences in SOR performance were observed, particularly for the hard trials.

#### Sample Phase

All animals explored during the sample phases for 25 sec in under 5 min on all trials. Analysis of the total time required to complete 25 sec of exploration in the sample phase yielded results comparable to the ZOR. That is, no significant age difference was observed, and no significant effect of condition (i.e., hard vs. easy), and no significant interaction (F < 1.26, p > 0.30 in all cases).

#### Choice Phase

During the first 2 minutes of the test trial (Fig. 1D), a significant main effect of condition (F_2,26_ = 4.34; p = 0.02) was observed. For all 3 age groups, discrimination index scores were significantly lower for hard discriminations than easy ones (p < 0.05 in all 3 cases). No significant effect of age was observed (F_2,26_ = 0.39; p = 0.68), however a significant age by difficulty interaction (F_2,26_ = 3.56; p = 0.04) demonstrated age-related differences in recognition under the hard condition. In fact, although aged animals were able to recognize the familiar object in the easy condition (t_10_ = 2.59; p = 0.01), they showed performance that was not significantly different from chance in the hard condition (t_10_ = 1.13; p = 0.14). Both young and middle-aged animals showed significant recognition in both easy (young: t_7_ = 3.38, p < 0.01; middle-aged: t_7_ = 3.51, p < 0.01) and hard (young: t_7_ = 2.43, p = 0.02; middle-aged: t_7_ = 2.21, p = 0.03) conditions.

### Comparison between Tasks

A direct comparison of performance in the hard condition between the tasks shows no significant effect of age (F_2,26_ = 2.21; p = 0.14). However, a significant effect of task (F_1,26_ = 4.94; p = 0.03) as well as a task by age interaction (F_1,26_ = 4.63; p = 0.04) were observed. This pattern is consistent with the interpretation that aged animals are impaired relative to their younger counterparts on the SOR and not ZOR.

### Correlations with Spatial Learning

The discrepancy in the results obtained from ZOR and SOR tasks suggests that age-related deficits reported in many previous studies of SOR may be the result of hippocampal contribution to task performance. Consistent with this hypothesis, comparison of recognition performance within both tasks with spatial learning in the same group of animals (Fig. 2C, D) reveals a significant correlation between performance on the probe trial in the MWM and the mean discrimination ratio obtained in the SOR (r = 0.75; p < 0.01). No significant correlation was seen between MWM performance and ZOR (r = 0.24, p = 0.31).

## DISCUSSION

The current results provide a potential explanation for the equivocal results seen among studies of age-related differences in recognition memory. This is because recognition memory performance *in the same group of older animals* can be made to appear normal or deficient as a result of small changes in the testing procedure. That is, when using procedures common to many studies that report age-related deficits in recognition memory (i.e., the use of an open field and the removal of animals during short delays between trials), older animals appear to have compromised memory, yet when these animals are tested using a variation of recognition memory testing (i.e., ZOR) that has been shown to make performance more resilient to hippocampal damage [27], recognition memory appears relatively intact. Importantly, the discrepancy in the data from ZOR and SOR cannot be explained either by individual differences in animals, or by features of the objects being tested because the same animals were tested in both conditions and the objects used were counterbalanced. Moreover, the fact that performance in the SOR and not ZOR is predicted by spatial memory performance in the MWM suggests that these differences are not merely the result of differences in the sensitivity of the ZOR and SOR tasks to the effects of ageing. Rather, it is more likely that these variations in testing procedure result in tasks with different cognitive demands.

As an alternative explanation, it could be hypothesized that order effects may cause the difference in performance in ZOR and SOR, as testing was not counterbalanced between groups. Such order effects could take two forms: one related to learning general task demands and one related to memory interference.

Since the ZOR and SOR have at least some similar task demands, it may be hypothesized that the experience from completing the ZOR and its similar task demands may provide some knowledge that can be transferred to improve performance on SOR. If younger animals benefit from this experience more than older ones, this may cause an age-related deficit to emerge as a result of testing order rather than true differences in the testing protocol. This explanation seems unlikely based on the data, however, since this should presumably cause the discrimination ratio for young animals to go up from the ZOR to the SOR as a result of the beneficial effect of previous experience. Instead, they decrease, which is inconsistent with this hypothesis.

It could also be proposed that the experience from completing the ZOR and its similar task demands may inherently make the later-tested SOR more difficult due to greater potential for interference from similar memories. Although one cannot completely rule out this possibility, this explanation also seems unlikely because 5 days passed between testing, and previous data suggests that older animals show steeper forgetting rates [34]. Based on this evidence, older animals should have an advantage in SOR under this circumstance, yet show a consistent deficit.

Finally, one could propose that visual or motor deficits could be responsible for the change in performance in SOR relative to ZOR, since older animals showed longer path lengths to reach visible platform locations in the MWM. The same objects were used (in a counterbalanced fashion) for the ZOR and SOR, so visual deficits cannot account for their poor performance selectively in SOR. Similarly, it is not clear how motor deficits could disproportionately impact performance in SOR. In fact, SOR should have less motor demands than ZOR, since animals can travel in a straight line between objects rather than cross the centre of a Y-maze. Thus, motor impairments should manifest as an age-related deficit in the opposite direction from the pattern observed here.

It remains a more parsimonious explanation that these variations in testing procedure between the ZOR and SOR result in tasks with different cognitive demands. It should be noted, however, that although the results presented here show proof of principle that the cognitive demands of these protocols are not equivalent, they do not provide insight into the nature of this difference, and this is a matter for future research. There are other several features that differ between these two paradigms that have the potential to influence performance on the ZOR and SOR tasks, such as perceptual and motor demands, number of trials and the interval among them, and the stress induced by each protocol. As such, the reason for this discrepancy can only be speculated upon based on the current data.

Among the possible explanations, it is plausible that SOR has a greater mnemonic demand relative to ZOR because of the delay inherent to the SOR. While the ideal comparison would be conducting SOR with zero delay, this was not feasible. In an effort to make the task as comparable as possible to the ZOR, the delay between the sample phase and the choice phase in the SOR was made as short as possible while still providing enough time to exchange the objects. However, this short delay may have been enough to engage the hippocampus, particularly in older animals. Consistent with this idea, a recent review [35] indicated that the delay between choice and testing phase was the single most reliable predictor of whether a hippocampal lesion would produce a deficit in performance in SOR. While this particular review suggested that 10 min was the minimum delay required to create a deficit in the absence of the hippocampus, it is possible that this threshold is lower for older animals if, for instance, the hippocampus is being engaged in these animals as a means to compensate for impairment in the function of other regions in the object recognition circuit such as the perirhinal cortex.

As an alternative, the delay interval and the change in the environment potentially provide conditions for SOR to tax working memory, and thus perhaps prefrontal cortex function, more than ZOR [36]. This hypothesis is consistent with the wealth of evidence for age-related deficits in working memory in multiple species [37], but is not consistent with the correlational data between performance in SOR and MWM. These structures show strong interdependence, however, and the hypotheses that these two tasks differentially tax the prefrontal cortex and hippocampus are not mutually exclusive.

Moreover, SOR may have a greater mnemonic demand than SOR as a result of using an open field. Although a rat may be capable of examining multiple objects simultaneously when they are presented together within an open field, this is not the typical pattern of behaviour. Rats explore each object independently before moving to explore the other object, often after a bout of exploring other parts of the environment [38]. Because rats explored only one object at a time, performance on the task necessarily requires memory to some extent [38].

The fact that performance in the best characterized test of hippocampal function (i.e., spatial memory performance in the MWM) predicts performance in the SOR is consistent with the proposal that (a) there is a hippocampal contribution to SOR performance in an open-field, and (b) it is this hippocampal contribution that disproportionately mediates age-related deficits in recognition memory as they are typically tested. Hippocampal involvement in SOR is controversial and well investigated [35,38,39], however, and the current data are neither causal nor definitive. Moreover, a wide array of human and animal data show that the function of the perirhinal cortex is impaired with age, and the current data do not undermine this wealth of evidence.

The current data do, however, demonstrate that some recognition testing protocols (e.g., in open field environments and/or with short delays) may significantly alter the observation of recognition deficits observed in older humans and animals. It is noteworthy that a recent meta-analysis on human recognition performance in normal ageing shows that the deficits observed show a similar variation on the basis of testing procedure [39]. Collectively, these results are compatible with the idea that multiple systems can drive performance in recognition tasks - a fast, automatic, hippocampus-independent familiarity system and a slower hippocampus-dependent recollection system [40,41]. Thus, the two protocols may tax highly overlapping but non-identical neural circuits that mediate recognition memory under different testing scenarios, with a circuit taxed by SOR that includes the hippocampus (and perhaps the prefrontal cortex), and a circuit taxed by ZOR that does not. Thus, relatively small changes in testing protocol can affect whether aged animals appear to have recognition memory deficits, consistent with a wealth of human literature [39].
